# Towards a fasting-mimicking diet for critically ill patients: the pilot randomized crossover ICU-FM-1 study

**DOI:** 10.1186/s13054-020-02987-3

**Published:** 2020-05-24

**Authors:** Lisa Van Dyck, Ilse Vanhorebeek, Alexander Wilmer, An Schrijvers, Inge Derese, Liese Mebis, Pieter J. Wouters, Greet Van den Berghe, Jan Gunst, Michaël P. Casaer

**Affiliations:** 1 Department of Cellular and Molecular Medicine, Clinical Division and Laboratory of Intensive Care Medicine, KU Leuven, Herestraat 49, B-3000 Leuven, Belgium; 2 Medical Intensive Care Unit, Department of Internal Medicine, University Hospitals Leuven, Leuven, Belgium

**Keywords:** Intensive care, Critical illness, Nutrition, Intermittent fasting, Metabolism, Autophagy

## Abstract

**Background:**

In two recent randomized controlled trials, withholding parenteral nutrition early in critical illness improved outcome as compared to early up-to-calculated-target nutrition, which may be explained by beneficial effects of fasting. Outside critical care, fasting-mimicking diets were found to maintain fasting-induced benefits while avoiding prolonged starvation. It is unclear whether critically ill patients can develop a fasting response after a short-term nutrient interruption. In this randomized crossover pilot study, we investigated whether 12-h nutrient interruption initiates a metabolic fasting response in prolonged critically ill patients. As a secondary objective, we studied the feasibility of monitoring autophagy in blood samples.

**Methods:**

In a single-center study in 70 prolonged critically ill patients, 12-h up-to-calculated-target feeding was alternated with 12-h fasting on day 8 ± 1 in ICU, in random order. Blood samples were obtained at the start of the study, at the crossover point, and at the end of the 24-h study period. Primary endpoints were a fasting-induced increase in serum bilirubin and decrease in insulin requirements to maintain normoglycemia. Secondary outcomes included serum insulin-like growth factor I (IGF-I), serum urea, plasma beta-hydroxybutyrate (BOH), and mRNA and protein markers of autophagy in whole blood and isolated white blood cells. To obtain a healthy reference, mRNA and protein markers of autophagy were assessed in whole blood and isolated white blood cells of 23 matched healthy subjects in fed and fasted conditions. Data were analyzed using repeated-measures ANOVA, Fisher’s exact test, or Mann–Whitney *U* test, as appropriate.

**Results:**

A 12-h nutrient interruption significantly increased serum bilirubin and BOH and decreased insulin requirements and serum IGF-I (all *p* ≤ 0.001). Urea was not affected. BOH was already increased from 4 h fasting onwards. Autophagic markers in blood samples were largely unaffected by fasting in patients and healthy subjects.

**Conclusions:**

A 12-h nutrient interruption initiated a metabolic fasting response in prolonged critically ill patients, which opens perspectives for the development of a fasting-mimicking diet. Blood samples may not be a good readout of autophagy at the tissue level.

**Trial registration:**

ISRCTN, ISRCTN98404761. Registered 3 May 2017.

## Background

For a long time, early artificial nutrition has been assumed to benefit critically ill patients, since prevention of a caloric deficit repeatedly has been associated with improved outcome [1–3]. However, recent large randomized controlled trials (RCTs) have shown that early enhanced feeding did not benefit critically ill patients, and two RCTs—the EPaNIC and PEPaNIC RCT—found that early supplementation of insufficient or contraindicated enteral nutrition by parenteral nutrition may even increase morbidity as compared to withholding parenteral nutrition until 1 week after intensive care unit (ICU) admission [4–9]. Hypothesis-generating, detailed, secondary analyses of both RCTs associated any feeding dose above the lowest dose with progressively increased harm, which suggests that the negative impact of early parenteral nutrition was dose-related and not related to the route of feeding [10, 11]. In line with this, two recent RCTs—the CALORIES and Nutrirea-2 RCT—found no outcome difference between the enteral and parenteral route of feeding when macronutrients were provided at isocaloric doses [12, 13]. Altogether, these data suggest that early enhanced nutritional support did not benefit critically ill patients and that relative macronutrient restriction may even be beneficial. Potential protective mechanisms of relative macronutrient restriction include activated autophagy and enhanced ketogenesis, among others, as put forward by experimental models and/or studies on human samples [14–17].

Although components of the fasting response may be beneficial in critical illness, prolonged starvation likely comes at a price [18]. A potential alternative to bring about fasting-induced benefits in critically ill patients is the design of a fasting-mimicking diet. Such diets substantially restrict food intake for several hours or days, which is alternated with periods of unlimited intake. Numerous animal studies in various models have demonstrated increased lifespan and protection against age-related diseases by implementation of such diet [19]. Interestingly, as compared to regular diets, fasting-mimicking diets were able to provide the full caloric target, while maintaining an improved metabolic profile during the period of full feeding. Since critical illness shows several parallels with normal aging, including increased cellular stress and accumulation of cellular damage, we hypothesized that a fasting-mimicking diet may be beneficial for critically ill patients as well [11, 20].

Until now, fasting-mimicking diets have not been tested in critically ill patients. Moreover, it remains unclear whether critically ill patients can develop a full metabolic fasting response. Indeed, ketogenesis may be impaired in critically ill patients, and the catabolic state induced by severe illness may preclude any additional fasting response [21–23]. Moreover, even if critically ill patients could develop a full fasting response, it remains unclear how long feeding should be restricted before such response would initiate. Although prolonged fasting intervals are theoretically more likely to induce a fasting response, a higher hourly feeding dose and associated fluid load would be needed to avoid underfeeding in such scenario. In this pilot crossover study, we investigated whether 12-h macronutrient restriction is sufficient to initiate a metabolic fasting response in prolonged critically ill patients. In healthy individuals, such response is characterized by increases in ketogenesis, serum bilirubin and urea, decreases in insulin concentrations and insulin-like growth factor I, and activation of autophagy [4, 19, 24–26]. Since it was unclear whether critically ill patients can initiate a full metabolic fasting response, the primary endpoints were based on fasting-induced alterations that were previously shown to be significantly impacted by withholding parenteral nutrition in the first week in ICU [4]. The study was meant as the first “dose-finding” step in the design of a fasting-mimicking diet for critically ill patients, to bring about an optimized metabolic profile and eventually to preserve cellular integrity through autophagy activation. If the pilot study would suggest feasibility, the subsequent aim would be to test such diet in a large RCT powered for clinical endpoints. In preparation of such future studies, we also investigated the feasibility of monitoring autophagy in blood samples as a less invasive surrogate for muscle biopsies.

## Methods

### Study design and participants

This study was a prospective, randomized, crossover pilot study performed in six medical/surgical ICUs at the University Hospitals of Leuven, Belgium. The study was performed in accordance with the principles of the Declaration of Helsinki. The Ethical Committee Research UZ/KU Leuven approved the protocol and consent forms (S59328). All patients or their legal representatives provided written informed consent before randomization. Patients were recruited from August 28, 2017, until July 11, 2018.

All adult patients (18 years or older) who were still in the ICU on day 6 were screened for eligibility. Patients were eligible if they were expected to stay in the ICU and dependent on both mechanical respiratory support and pharmacological and/or mechanical hemodynamic support for at least another 72 h. Patients were excluded if they had serum bilirubin concentrations above 5 mg/dl, were enrolled in another RCT, were considered moribund or had formal therapy restriction as part of advanced care planning, were pregnant or lactating, had any oral intake, were previously enrolled in the study, or were unable to be fasted or fed for 12 consecutive hours (e.g., due to specific need for glucose-containing infusions or due to procedure-related fasting). After having obtained informed consent on day 7 ± 1, patients still meeting the inclusion criteria were randomized to receive 50% of their calculated daily caloric target over 12 consecutive hours, followed by 12-h fasting (feeding-fasting) on day 8 ± 1, or vice versa (fasting-feeding). Day 8 ± 1 was chosen as the intervention day, as our common practice involves withholding parenteral nutrition in the first week in ICU, implying relative macronutrient restriction in the acute phase of illness. Thereby, inclusion of patients in the first week in ICU would have led to a minor difference in caloric intake between the fasting and feeding interval, which would have inflated the chance of false-negative results. Moreover, if the project would ultimately show that fasting-mimicking diets are beneficial, this would be particularly relevant for patients with prolonged ICU stay. Patients were stratified according to admission diagnosis (cardiac surgery versus others) and randomization was performed with use of a central computer in permuted blocks of 10 per stratum. The block size was unknown to the treating physicians and nurses.

During the first week in ICU, all patients received enteral nutrition if possible, which was increased according to tolerance. No parenteral nutrition was administered in the first week after ICU admission as per the centers’ nutrition protocol. During ICU stay, all patients received intravenous supplements of trace elements and vitamins, which was continued until the patient received the full calculated caloric target through EN and oral intake (Additional table 1). Blood phosphate, potassium, and magnesium were monitored daily, in order to allow prevention and/or early detection of deficiencies and refeeding syndrome. All patients received blood glucose control targeting 80–110 mg/dl with insulin throughout ICU stay until they were able to eat by mouth. To that purpose, arterial blood glucose concentrations were monitored every 1 to 4 h with a blood gas analyzer, and insulin dose was adjusted through continuous intravenous infusion.

On the intervention day at day 8 ± 1, during the 12-h feeding interval, patients received artificial nutrition to cover 100% of their hourly calculated caloric goal. Nutrition primarily consisted of enteral nutrition, but was supplemented with parenteral nutrition if enteral nutrition was insufficiently tolerated or contraindicated. The enteral and parenteral nutrition formulas prescribed at the discretion of the treating physician are listed in Additional table 2. The nutritional target was calculated and based on corrected ideal body weight, age, and gender (Additional table 1). During the 12-h fasting interval, artificial nutrition was stopped, including any infusion of glucose-containing maintenance infusions. An exception was made for calories from propofol and intravenously administered drugs requiring mandatory dilution in glucose. The insulin infusion rate was preemptively reduced at the start of the 12-h fasting interval to reduce the risk of hypoglycemia. Patients received non-glucose containing fluids in order to provide adequate hydration at the discretion of the treating physician. If a patient developed hypoglycemia, a glucose bolus and/or a glucose-containing maintenance infusion were given at the discretion of the treating physician.

At the start of the study intervention (8:00 h), at the crossover point after 12 h (20:00 h), and at the end of the intervention (after 24 h, at 8:00 h the following morning), an arterial blood sample was taken. Beta-hydroxybutyrate (BOH) concentrations were measured at the bedside every 4 h using a point-of-care ketometer (StatStrip Glucose/Ketone Xpress2® Meter, Nova Biomedical, provided by Menarini Diagnostics).

To obtain a healthy reference range for autophagic markers in white blood cells and whole blood, healthy volunteers were recruited with similar age, gender, and BMI distributions as the patients (Table 1). Volunteers were instructed to fast for at least 12 h overnight prior to blood sampling in the morning (fasted state). A second blood sample was obtained 2 h after a copious breakfast (fed state).

**Table 1:** Baseline characteristics of participants

Baseline characteristic	Fasting–feeding *N* = 35	Feeding–fasting *N* = 35	Controls *N* = 23
Age**—**median [IQR]	63.2 [51.5–72.8]	67.7 [57.6–74.4]	69.2 [55.8–79.4]
Male gender**—***n* (%)	18 (51.4)	18 (51.4)	10 (43.5)
BMI**—**median [IQR]	25.2 [23.3–31.6]	24.6 [21.7–27.1]	27.3 [24.3–29.4]
Admission to surgical ICU**—***n* (%)	22 (62.9)	22 (62.9)	NA
Cardiac surgery**—***n* (%)	7 (20.0)	6 (17.1)	NA
Emergency admission**—***n* (%)	32 (91.4)	31 (88.6)	NA
Sepsis upon ICU admission**—***n* (%)	19 (54.3)	20 (57.1)	NA
APACHE II**—**median [IQR]	33 [27–35]	31 [25–36]	NA
NRS score**—**median [IQR]	4 [3–5]	4 [4–5]	NA
Diabetes**—***n* (%)	7 (20.0)	8 (22.9)	3 (13.0)
History of malignancy**—***n* (%)	10 (28.6)	10 (28.6)	3 (13.0)
Pre-admission dialysis**—***n* (%)	2 (5.7)	0 (0.0)	0 (0.0)
Study day**—**median [IQR]	8 [8–10]	8 [8–9]	NA
SOFA score on randomization day**—**median [IQR]	8 [6–10]	7 [6–11]	NA

*Abbreviations*: *IQR* interquartile range, *BMI* body mass index, *ICU* intensive care unit, *APACHE* Acute Physiology and Chronic Health Evaluation, *NRS* Nutritional Risk Screening, *SOFA* Sequential Organ Failure Assessment

### Data collection

We collected demographical data and daily records of clinical parameters and treatments from the patient data management system (MetaVision Suite, iMDsoft). Upon ICU admission, we quantified severity of illness according to the Acute Physiology and Chronic Health Evaluation II score (APACHE II), and nutritional risk according to the Nutritional Risk Screening score (NRS), and scored sepsis according to the Sepsis-3 criteria [27]. To quantify severity of illness at inclusion in the study, the Sequential Organ Failure Assessment (SOFA) score was calculated over the 24 h preceding the intervention day. On the intervention day, we collected detailed data regarding nutrition, blood glucose, insulin requirements, and propofol administration in each 12-h period. Energy from parenteral nutrition included total calories from parenteral nutrition solutions, glucose-containing fluids, and propofol. Severe hypoglycemia was defined as arterial blood glucose concentration below 40 mg/dl.

We recorded the development of new infections and the persistent need for hemodynamic support, respiratory support and renal replacement therapy at day 7 after randomization or at ICU discharge if patients were discharged earlier. Hemodynamic support was defined as mechanical or pharmacological (epinephrine, norepinephrine, dobutamine, dopamine, or vasopressin in any dose) support, and mechanical respiratory support was defined as any ventilation method with positive pressure generation. Patients who died within 7 days after the intervention were marked as persistently dependent on hemodynamic and respiratory support. We recorded mortality within 7 days after randomization, ICU mortality, and mortality within 90 days after randomization. The cause of death was recorded for all patients who died within 90 days after randomization.

### Outcome measures

The primary endpoints were a fasting-induced increase in total serum bilirubin and a decreased insulin need to maintain normoglycemia. The choice of the primary endpoints was based on well-documented fasting-associated alterations in healthy individuals and in critically ill patients in whom parenteral nutrition was withheld until 1 week after ICU admission [4, 18, 25]. Secondary outcomes were changes in plasma and blood ketone concentrations, in serum insulin-like growth factor (IGF-I), and in serum urea. These parameters are similarly affected by short-term fasting in healthy humans, except for urea that shows slower kinetics [18, 19, 26]. Tertiary, exploratory endpoints included the mRNA and protein expression of commonly used autophagic markers in whole blood and isolated white blood cells.

### Lab analyses

Total serum bilirubin was measured with a commercially available assay (Total Bilirubin Reagent, ThermoFisher) and standards with known bilirubin concentrations (Bilirubin Standard Kit, Verichem). Serum urea and plasma beta-hydroxybutyrate (BOH) were measured with commercially available colorimetric assays (Urea Assay Kit, Cell Biolabs Inc.; and EnzyChrom Ketone Body Assay kit, BioAssay Systems). Serum IGF-I was measured with a commercially available enzyme-linked immunosorbent assay (Human IGF-I Quantikine ELISA kit, R&D Systems).

Markers of autophagy were investigated in whole blood and in isolated white blood cells. Whole blood was collected in PAXgene™ Blood RNA tubes (PreAnalytiX) and was stored at − 80 °C until RNA extraction with PAXgene™ Blood RNA kits (PreAnalytiX). White blood cells were isolated within 2 h after blood sampling. Red blood cells were first removed via an osmotically active lysis buffer (NH_4_Cl 8.29 g/l, EDTA disodium 2-H_2_O 0.0372 g/l, KHCO_3_ 1 g/l). Subsequently, white blood cells were lysed and stored at − 80 °C until simultaneous extraction of RNA and proteins with NucleoSpin® RNA/protein filters (Macherey-Nagel). Four hundred fifty nanograms of mRNA of the PAXgene tubes and 210 ng mRNA of the isolated white blood cells were reverse transcribed to cDNA. Real-time PCR (StepOne Plus, Applied Biosystems) for gene expression analysis was performed with use of TaqMan chemistry (Applied Biosystems, Hs00223937_m1 for *atg3*, Hs00169468_m1 for *atg5*, Hs00893766_m1 for *atg7*, Hs00177654_m1 for *p62*). Relative gene expression was determined with the 2−ΔΔCt method using *beta*-*2 microglobulin* (*B2M*, Hs00187842_m1) as housekeeping gene. Western blotting was performed on proteins extracted from isolated white blood cells with primary antibodies against LC3 (Sigma, L7543) and p62 (Novus Biologicals, H00008878-M01) and against actin (Abcam, ab3280) as housekeeping protein. Secondary horseradish peroxidase-conjugated antibodies were purchased from DakoCytomation. Blots were visualized with the G:BOX Chemi XRQ (SynGene) and analyzed with SynGene software. Both real-time PCR and western blot data were expressed relative to the median of the 23 fed healthy controls.

### Statistical analyses

We calculated that a sample size of 70 patients, in a crossover design using repeated measures ANOVA, with an alpha level of 0.05, would be able to detect a 0.3 mg/dl increase in serum bilirubin concentrations with 93% power and a decrease of 40 IU per 24 h in insulin requirements to maintain normoglycemia with 99% power [28]. The expected effect size and total variance for the primary endpoints (*f* = 0.169 for serum bilirubin, *f* = 0.3055 for insulin requirements) were based on changes observed in the EPaNIC RCT [4], during which nutrition was abruptly increased from day 7 to day 8 in a subset of patients.

Variables were summarized as frequencies and percentages, medians and interquartile ranges, or means and standard errors of the mean, as appropriate. Data were compared with use of repeated measures ANOVA, Fisher’s exact test, or Mann–Whitney *U* test, as appropriate. Where needed, data were transformed with a square root to obtain a near-normal distribution. All statistical analyses were performed with JMP® Pro (v14.0.0, SAS Institute). Power was calculated in G*Power (version 3.1.9, Heinrich Heine Universität Düsseldorf). Two-sided *p* values ≤ 0.05 were considered to indicate statistical significance, without correction for multiple comparisons.

## Results

### Patients and intervention

**Fig. 1:**
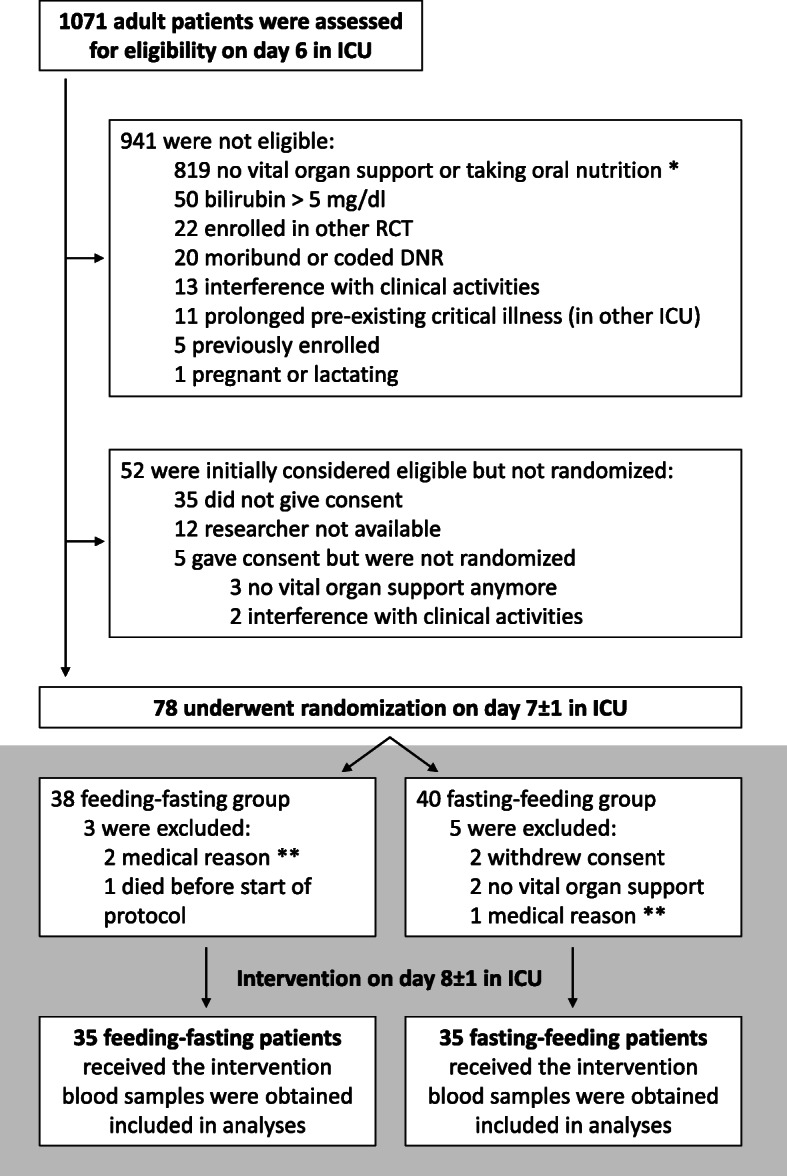
Enrollment and randomization. Reasons for ineligibility and non-inclusion of eligible patients are listed. Ultimately, 35 patients per randomization group completed the study protocol, hence 70 patients in total. *Vital organ support was defined as dependency on mechanical ventilation and mechanical and/or pharmacological hemodynamic support. **Medical reasons include high glucose need to treat hypernatremia (*n* = 2) or multiple hypoglycemic episodes between randomization and start of the protocol (*n* = 1). ICU, intensive care unit; RCT, randomized controlled trial; DNR, do not resuscitate

**Fig. 2:**
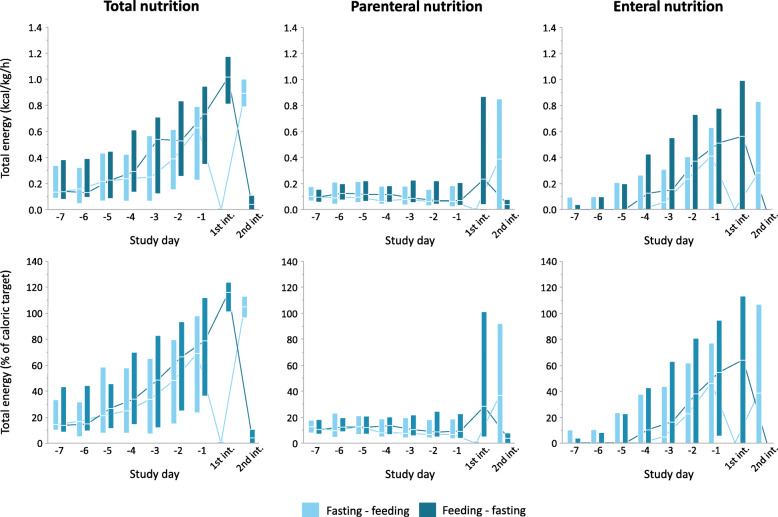
Calories administered. Daily total calorie administration and calories administered via the parenteral and enteral route separately are shown for both randomization groups starting 7 days before the intervention day. On the intervention day, caloric intake is shown for the two intervention intervals separately. In the top panels, administered calories are expressed as kilocalories per kilogram of body weight per hour. In the bottom panels, administered calories are expressed relative to the caloric target. Kilocalories from parenteral nutrition include kilocalories from total parenteral nutrition (glucose, proteins, and lipids), infused glucose-containing fluids, and propofol. Boxes represent interquartile ranges, and horizontal lines within the boxes represent the medians. Lines connect the medians from subsequent days or study intervals. Int., interval

On day 6, 1071 patients were screened for eligibility. Reasons for exclusion are shown in Fig. 1. On day 8 ± 1, 70 randomized patients received the crossover intervention (*n* = 35 in each group). Baseline characteristics and calculated caloric targets were comparable for both groups (Table 1 and Additional table 3). Over the entire intervention day, patients received similar amounts of total calories (*p* = 0.65), consisting of similar amounts of enteral (*p* = 0.82) and parenteral calories (*p* = 0.30) (Fig. 2 and Additional table 3). During the 12-h feeding interval, patients received a median of 0.95 kcal/kg/h (interquartile range (IQR) 0.80–1.15 kcal/kg/h) and during the 12-h fasting interval 0.03 kcal/kg/h (IQR 0.00–0.06 kcal/kg/h). Patients of both randomization groups received comparable amounts of calories during the feeding interval (*p* = 0.11), whereas during the fasting interval, patients in the feeding-fasting group received slightly more calories with a median of 0.04 kcal/kg/h (IQR 0.00–0.11 kcal/kg/h) as compared to the fasting-feeding group with a median of 0.00 kcal/kg/h (IQR 0.00–0.01 kcal/kg/h) (*p* = 0.0004). In the total study population, administered calories corresponded to 108.1% of the calculated caloric target (IQR 99.0–119.9%) during the feeding interval and 0.3% of the calculated caloric target (IQR 0.0–7.2%) during the fasting interval (Fig. 2). Over the entire study day, patients reached 55.8% of their calculated caloric target (IQR 51.2–62.8%).

### Metabolic response to 12-h fasting versus feeding

**Fig. 3:**
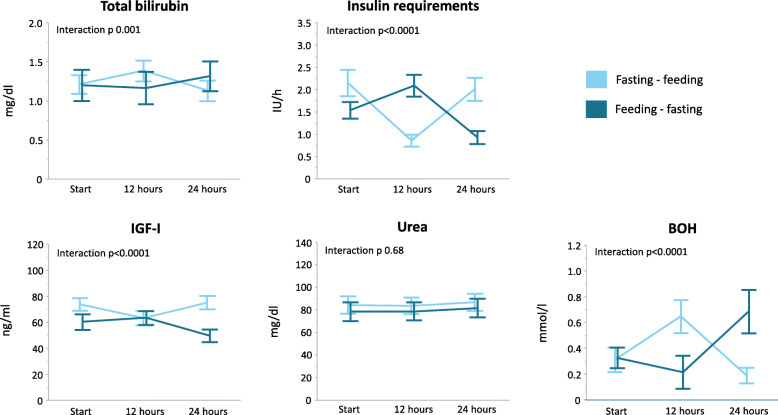
Metabolic fasting parameters. Results of serum total bilirubin, insulin requirements to maintain normoglycemia, serum urea, serum IGF-I, and plasma BOH are shown on the study day for both randomization groups. For bilirubin, urea, IGF-I, and BOH measurements, blood samples were obtained at the start of the protocol, at the end of the first study interval, and at the end of the second study interval. For insulin requirements, insulin administration was averaged per hour for the 24 h before the study day to obtain a baseline value and averaged per hour for both study intervals. Reported *p* values were obtained with repeated measures ANOVA. Lines represent means, and bars represent standard errors of the means. IGF-I, insulin-like growth factor I; BOH, beta-hydroxybutyrate

Both serum total bilirubin and insulin requirements were significantly affected by the intervention (interaction *p* = 0.002 for serum total bilirubin, interaction *p* < 0.0001 for insulin requirements, Fig. 3). Regardless of randomization for order, 12-h fasting increased serum total bilirubin with a mean of 0.20 ± 0.06 mg/dl (*p* = 0.0004) and decreased insulin requirements with a mean of 1.16 ± 0.14 IU/h (*p* < 0.0001) as compared with feeding. Over the total intervention day, the cumulative insulin dose was similar between both groups (*p* = 0.15, Additional table 3).

Plasma BOH and serum IGF-I concentrations were significantly affected by the intervention (both interaction *p* < 0.0001). A 12-h fasting period increased BOH with a mean of 0.47 ± 0.07 mmol/l (*p* < 0.0001) and decreased IGF-I with a mean of 13.9 ± 1.6 ng/ml (*p* < 0.0001) as compared with the 12-h feeding period. Serum urea was unaffected by the intervention (interaction *p* = 0.68).

**Fig. 4:**
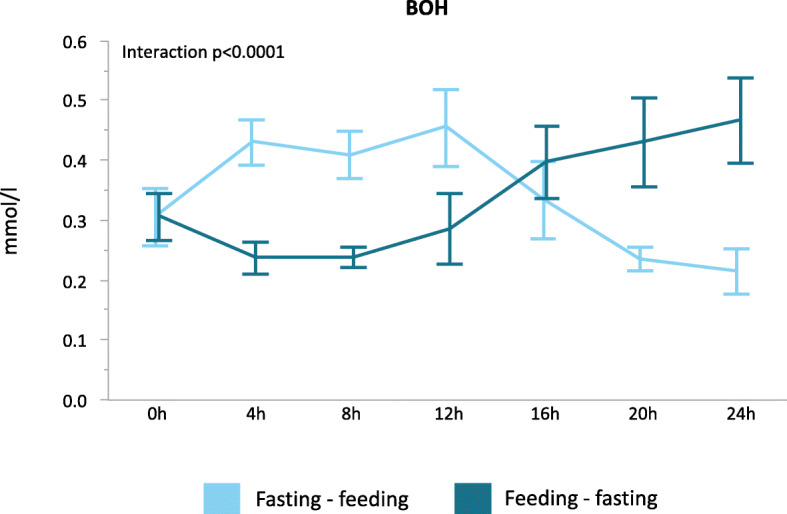
POC ketone meters. Results of BOH measurements with use of a point of care ketone meter during the study day. BOH was measured every 4 h. Results are shown for both randomization groups. 0 h marks the start of the study day, 12 h marks the end of the first study interval, and 24 h marks the end of the second study interval. The reported *p* value was obtained using repeated measures ANOVA. Lines represent means, and bars represent standard errors of the means. BOH, beta-hydroxybutyrate

Consecutive point-of-care BOH measurements confirmed a significantly different time profile for both randomization groups (interaction *p* < 0.0001). BOH was already increased from 4 h fasting onwards and was suppressed from 4 h feeding onwards (mean delta BOH after 4 h of fasting 0.1 ± 0.37 mmol/l, after 4 h of feeding − 0.1 ± 0.23 mmol/l, *p* = 0.0001, Fig. 4). BOH remained elevated throughout the 12-h fasting interval (mean BOH after 4 h of fasting 0.4 ± 0.04 mmol/L, after 8 h of fasting 0.4 ± 0.04 mmol/l, after 12 h of fasting 0.5 ± 0.05 mmol/l). As compared to 4 h fasting, BOH did not significantly change over the remainder of the 12-h fasting interval (mean delta between 4 h and 8 h of fasting 0.0 ± 0.04 mmol/l, mean delta between 4 h and 12 h of fasting 0.0 ± 0.05 mmol/l, *p* 0.76).

### Autophagy activation in whole blood and white blood cells

**Fig. 5:**
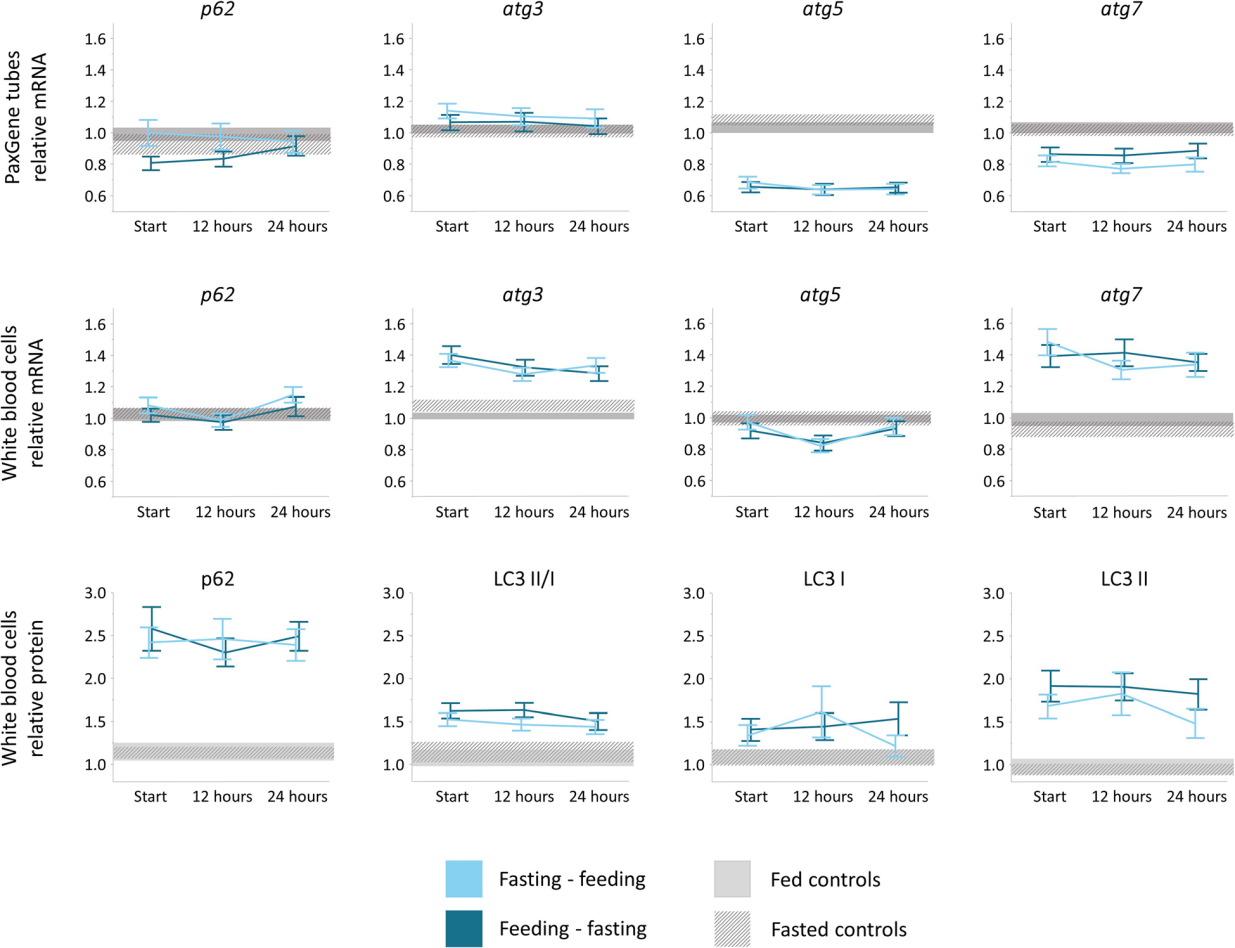
Markers of autophagy. mRNA and protein expression of autophagy-related genes in whole blood and isolated white blood cells are shown for healthy controls and patients. Results are expressed relatively to the median of 23 fed healthy controls. Patient results are shown as lines representing the means and bars representing the standard errors of the means. For controls, the grey areas represent the standard errors of the means in fed (solid grey) and fasted (patterned grey) conditions. Interaction *p* values are higher than 0.05 for all studied genes (see the “Results” section for exact *p* values)

mRNA expression of autophagy-related genes was not significantly affected by the intervention, neither in whole blood (interaction *p* 0.08 for *p62*, 0.75 for *atg3*, 0.51 for *atg5*, and 0.64 for *atg7*), nor in isolated white blood cells (interaction *p* 0.62 for *p62*, 0.17 for *atg3*, 0.29 for *atg5*, and 0.13 for *atg7*) (Fig. 5). Also in healthy volunteers, there was no effect of nutrition on gene expression in whole blood when comparing the fasted state to the fed state (*p* values for within-subject effect of time 0.40 for *p62*; 0.58 for *atg3*; 0.07 for *atg5*; 0.58 for *atg7*). In the isolated white blood cells of controls, fasting slightly induced expression of *atg3* with an increase of 6.7%, but no effect was observed on the other autophagy genes (*p* values for within subjects effect of time 0.83 for *p62*; 0.04 for *atg3*; 0.94 for *atg5*; 0.30 for *atg7*).

Protein expression of key autophagy markers was not significantly affected by the intervention (interaction *p* 0.38 for p62, 0.56 for the ratio of LC3 II/LC3 I, 0.12 for LC3 I, and 0.37 for LC3 II) (Fig. 5). Also in healthy volunteers, protein levels of these markers were not different between the fasted and the fed state (*p* values for within subjects effect of time 0.91 for p62, 0.46 for the ratio of LC3 II/LC3 I, 0.52 for LC3 I, and 0.63 for LC3 II).

### Morbidity and mortality

During the 12-h fasting interval, severe hypoglycemia occurred in 3 patients. All events occurred under treatment with intravenous insulin; hence, no patient suffered from spontaneous hypoglycemia. In two of the events, unlike prescribed in the study protocol, the insulin dose was not reduced at the start of the fasting interval. All hypoglycemic events were countered by parenteral glucose administration. None of the patients died within 24 h after the hypoglycemic event. In the first week after the intervention, the incidence of new infections and persistent need for hemodynamic support, mechanical respiratory support, and renal replacement therapy were comparable between both randomization groups (Additional table 3). Mortality within the first week after the intervention and ICU mortality were comparable between both randomization groups. Ninety-day mortality was higher in the feeding-fasting group than in the fasting-feeding group (*p* 0.003). Causes of death are reported in Additional table 4.

## Discussion

In this randomized crossover study of 70 prolonged critically ill patients, we found that 12-h nutrient restriction initiated a metabolic fasting response, as evidenced by a rise in serum total bilirubin and a decrease in insulin needs to maintain normal blood glucose concentrations. Also, a rise in plasma BOH and a decrease in serum IGF-I was observed with a 12-h fast, whereas serum urea was unaffected. The increase in blood BOH was already present after 4 h of fasting, and prolongation of the fasting period did not result in a further increase. Neither in healthy controls nor in prolonged critically ill patients did a 12-h fasting affect autophagy-related gene and protein expression in whole blood or isolated white blood cells.

Recent RCTs have shown that accepting an early macronutrient deficit by withholding PN in the acute phase of critical illness may be beneficial, which suggests that activating a fasting response may be protective in critically ill patients [4, 5]. Beyond the acute phase, however, prolonged starvation may support ongoing catabolism, which is unwanted. Recent studies in other models have suggested that fasting-induced benefits can also be brought about by so-called fasting-mimicking diets, which alternate periods of restricted feeding with periods of unlimited feeding [19]. Indeed, in healthy adults and mice, fasting-mimicking diets improved the metabolic profile, inflammation, and regenerative markers, which in animal models also prevented age-related diseases and increased lifespan [19]. Interestingly, in an animal model, also in the fed phase of the fasting-mimicking diet, ketone concentrations were higher than with the ad libitum diet, which suggests that fasting responses could be partially maintained in the fed state [19]. The current study opens perspectives for the further design and clinical validation of a fasting-mimicking diet for critically ill patients. Indeed, our pilot study demonstrated that 12-h feeding interruption initiated a metabolic fasting response in prolonged critically ill patients, with increases in serum bilirubin and blood/plasma BOH and decreases in insulin need and IGF-I. Although the observed differences were relatively small, fasting-mimicking diets in other settings have shown similarly small but sustained changes while providing clinical benefit [19, 29]. In our study, serum urea was not affected, which may be explained by the relatively brief fasting period. Indeed, in healthy adults, urea concentrations only rose after a considerably longer fasting episode [18]. On the other hand, urea may not be a good marker of a metabolic fasting response in critically ill patients, in whom artificial feeding may also result in increased ureagenesis through catabolism of the supplementary provided amino acids [30]. Future studies should investigate whether time-restricted feeding by daily interruption of artificial feeding for 12 h can maintain a sustained metabolic fasting response and whether this allows to induce autophagy in tissues of interest and improves clinical outcomes. Although speculative, another potential advantage of time-restricted feeding could be a better preservation of circadian rhythm when the fasting interval would be held during the night [31, 32]. Time-restricted feeding also has potential downsides, however, including an increased risk of prolonged underfeeding if nutrition cannot be proportionally increased in the feeding interval or else a potentially increased risk of feeding intolerance, and potentially larger fluctuations in blood glucose, which have been associated with worse outcome [33, 34].

In contrast to previous studies suggesting impaired ketogenesis in critical illness, our results indicate increased ketogenesis already after 4 h of fasting [22, 23]. Although speculative, prevention of hyperglycemia in our study may have played a role. Indeed, high circulating glucose concentrations can suppress lipolysis, and this condition was prevented by continuous intravenous insulin administration [35]. On the other hand, insulin also suppresses lipolysis and ketogenesis [36]. However, our group previously demonstrated in a subanalysis of a RCT that tight glucose control improved insulin sensitivity in skeletal muscle of prolonged critically ill patients, but not in liver [37]. Hence, in prolonged critically ill patients, tight glucose control was realized with similar serum insulin concentrations as in patients in whom moderate hyperglycemia was tolerated, in the presence of severe hepatic insulin resistance.

In both healthy volunteers and prolonged critically ill patients, we did not find any consistent impact of a 12-h fasting episode on gene and protein expression of autophagy-related genes in whole blood and isolated white blood cells. This contrasts with a previous study that showed activation of autophagy in skeletal muscle by withholding PN until 1 week after ICU admission, which correlated with improved muscle function [14]. This apparent discrepancy may point to tissue-dependent changes in autophagy activation. Indeed, in mice, starvation-induced autophagy activation differed among organs, with a more distinct early induction in muscle as compared to other tissues [38]. Furthermore, the metabolism of white blood cells has been shown to be relatively resistant to starvation in healthy adults [39]. Hence, our data may suggest that blood samples are not a good surrogate for autophagy at the level of other tissues, although this requires further investigation in a study that compares different tissues. Alternatively, the fasting interval may have been too short to detect fasting-induced autophagy. However, we also did not find any impact of fasting on autophagic markers in healthy volunteers. Moreover, autophagy has been shown to be activated by 12-h fasting in muscle of healthy animals, although one should be cautious when extrapolating results from animals to humans [40].

Unexpectedly, the study revealed a significantly different 90-day mortality between both randomization groups. Although we cannot exclude an impact of randomization, it appears unlikely that a feeding study only lasting 24 h would affect mortality on a longer term, as none of the large RCTs on nutritional management in the ICU has observed any impact of nutrition on survival [33]. Moreover, short-term morbidity and mortality at day 7 and in ICU were similar in both groups. Finally, since all patients received the same interventions in a crossover design at day 8 in ICU, it seems unlikely that the order of such intervention would have affected clinical outcome only on the long term. Instead, it could be that patients in the feeding-fasting group may have been sicker than the fasting-feeding group, although this was not captured by the SOFA scores.

The study has important strengths. We demonstrated a clear initiation of a metabolic fasting response during a 12-h fasting period, which was consistently observed on different metabolic parameters in a crossover design. Our study inherently has some limitations. The sample size was relatively small, and the study may have been underpowered for some secondary endpoints. Second, we only investigated a 12-h fasting interval, whereby we do not have data on longer fasting intervals that may induce a more pronounced metabolic fasting response. However, the duration of fasting was a pragmatic choice between a sufficiently long period of fasting while leaving an acceptable time to feed. Third, differences in concomitant medication between the two study periods may theoretically have impacted certain outcome parameters. However, this risk is minimal in a crossover study, since we expect that significant medication adaptations on the intervention day with immediate impact on the studied parameters would be rare. Fourth, we calculated the daily caloric target using a formula. Although common practice, this value does not necessarily match the energy expenditure measured by indirect calorimetry. Finally, to reduce the metabolic burden of administering the daily calculated nutrient dose over only 12 h, we only administered half of the calculated daily nutrition dose in the 12-h feeding interval.

## Conclusion

A 12-h macronutrient interruption was able to initiate a metabolic fasting response in prolonged critically ill patients, with increases in bilirubin and ketones and decreases in insulin needs and IGF-I. Twelve hours fasting did not affect markers of autophagy in isolated white blood cells and whole blood samples of prolonged critically ill patients or healthy volunteers, and thus, blood samples may not be a good readout to assess autophagy at the level of other tissues. The current study opens perspectives for the development of a fasting-mimicking diet for critically ill patients, which could consist of repeated blocks of 12 h of feeding alternated with 12 h of fasting. Future research should investigate whether such diet is able to maintain a sustained fasting response and whether this delivers clinical benefit.

## Supplementary information

**Additional file 1.** (VanDyck-ICU-FM-additional_table1). Calculation of caloric target. Description of data: Formula used to calculate the caloric target.

**Additional file 2.** (VanDyck-ICU-FM-additional_table2). Formulas of enteral and parenteral nutrition. Description of data: Formulas of enteral and parenteral nutrition used in the study.

**Additional file 3.** (VanDyck-ICU-FM-additional_table3). Nutrition on the intervention day and clinical endpoints. Description of data: Nutrition on the intervention day combined for both intervention windows. ICU-related complications for 7 days after the study day, or until ICU discharge if the ICU stay was shorter, and for short- and long-term mortality.

**Additional file 4.** (VanDyck-ICU-FM-additional_table4). Cause of death for 90 day mortality. Description of data: Cause of death for patients who died within 90 days after randomization

## Data Availability

Data sharing will be considered only on a collaborative basis with the principal investigators, after evaluation of the proposed study protocol and statistical analysis plan.

## Abbreviations

APACHE II: Acute Physiology and Chronic Health Evaluation II
BMI: Body mass index
BOH: Beta-hydroxybutyrate
EN: Enteral nutrition
ICU: Intensive care unit
IGF-I: Insulin-like growth factor I
NRS: Nutritional Risk Screening
PN: Parenteral nutrition
POC: Point of care
RCT: Randomized controlled trial
SOFA: Sequential Organ Failure Assessment
