# Supplementary material for: Towards a fasting-mimicking diet for critically ill patients: the pilot randomized crossover ICU-FM-1 study
Source: Crit Care. 2020 May 24;24:249. doi: 10.1186/s13054-020-02987-3 (PMC7245817; doi:10.1186/s13054-020-02987-3)
Supplement: Supplementary file 1 — Additional file 1. (VanDyck-ICU-FM-additional_table1). Calculation of caloric target. Description of data: Formula used to calculate the caloric target. [file 13054_2020_2987_MOESM1_ESM.docx]

**Additional table 1: Calculation of caloric target**

| **Caloric target = caloric need x corrected ideal body weight** | |
| --- | --- |
| **Formula for calculating ideal body weight (IBW)** | |
| Female patient | 45.5 + [0.91 x (height in cm – 152.4)] |
| Male patient | 50 + [0.91 x (height in cm – 152.4)] |
| **Corrected ideal body weight** |  |
| If BMI < 18.5 | (IBW + actual body weight) / 2 |
| If 18.5 ≤ BMI ≤ 27 | IBW |
| If BMI > 27 | IBW x 1.2 |
| **Caloric need (kcal/kg/day)** |  |
|  | Female patient Male patient |
| Age ≤ 60 years | 24 30 |
| Age > 60 years | 20 24 |

Abbreviations: IBW: ideal body weight, BMI: body mass index.
