# Supplementary material for: Towards a fasting-mimicking diet for critically ill patients: the pilot randomized crossover ICU-FM-1 study
Source: Crit Care. 2020 May 24;24:249. doi: 10.1186/s13054-020-02987-3 (PMC7245817; doi:10.1186/s13054-020-02987-3)
Supplement: Supplementary file 2 — Additional file 2. (VanDyck-ICU-FM-additional_table2). Formulas of enteral and parenteral nutrition. Description of data: Formulas of enteral and parenteral nutrition used in the study. [file 13054_2020_2987_MOESM2_ESM.docx]

**Additional table 2: Formulas of enteral and parenteral nutrition**

| **Enteral nutrition** | **Parenteral nutrition** |
| --- | --- |
| Isosource Standard (Nestlé SA, Switzerland) | Olimel N7E (Baxter International Inc., United States) |
| Isosource Energy (Nestlé SA, Switzerland) | Clinimix N14 (Baxter International Inc., United States) |
| Isosource Fibre (Nestlé SA, Switzerland) | Clinimix N17 (Baxter International Inc., United States) |
| Isosource Energy Fibre (Nestlé SA, Switzerland) | Smoflipid (Fresenius Kabi, Germany) |
| Nutrison Concentrated (Nutricia, The Netherlands) | In-house composed fluid-restricted PN formula |
| Peptamen (Nestlé SA, Switzerland) |  |

Enteral and parenteral nutrition formulas were described at the discretion of the treating physician.
