# Supplementary material for: Towards a fasting-mimicking diet for critically ill patients: the pilot randomized crossover ICU-FM-1 study
Source: Crit Care. 2020 May 24;24:249. doi: 10.1186/s13054-020-02987-3 (PMC7245817; doi:10.1186/s13054-020-02987-3)
Supplement: Supplementary file 3 — Additional file 3. (VanDyck-ICU-FM-additional_table3). Nutrition on the intervention day and clinical endpoints. Description of data: Nutrition on the intervention day combined for both intervention windows. ICU-related complications for 7 days after the study day, or until ICU discharge if the ICU stay was shorter, and for short- and long-term mortality. [file 13054_2020_2987_MOESM3_ESM.docx]

**Additional table 3: Nutrition on the intervention day and clinical endpoints**

|  | **Fasting – Feeding**  **N = 35** | **Feeding – Fasting**  **N = 35** | **p value** |
| --- | --- | --- | --- |
| **Nutrition on the intervention day** |  |  |  |
| Calculated caloric target | 1641 [1258-1954] | 1476 [1258-1650] | 0.15 |
| Total calories study day– median [IQR] |  |  |  |
| Kcal/day | 840 [705-1021] | 857 [708-1031] | 0.65 |
| Kcal/kg/day | 10.8 [9.6-12.0] | 12.9 [10.6-15.4] | 0.02 |
| Total insulin dose study day (IU) – median [IQR] | 30 [14-52] | 31 [17-50] | 0.70 |
| **Outcome** |  |  |  |
| New infection – n (%) | 13 (37.1) | 19 (54.3) | 0.23 |
| Persistent hemodynamic support – n (%) | 16 (45.7) | 19 (54.3) | 0.63 |
| Persistent respiratory support – n (%) | 18 (51.4) | 26 (74.3) | 0.08 |
| Persistent renal replacement therapy – n (%) | 5 (14.3) | 12 (34.3) | 0.09 |
| Mortality 7 days after study day – n (%) | 6 (17.1) | 7 (20.0) | >0.99 |
| ICU mortality – n (%) | 9 (25.7) | 16 (45.7) | 0.13 |
| 90-day mortality – n (%) | 10 (28.6) | 23 (65.7) | 0.003 |

Nutrition on the intervention day was calculated by combining data on nutrition for the separate intervention windows. The patients’ hospital records were screened for the development of ICU-related complications for 7 days after the study day, or until ICU discharge if the ICU stay was shorter, and for short- and long-term mortality.

Abbreviations: IQR: interquartile range, IU: international units, ICU: intensive care unit.
