# Supplementary material for: Towards a fasting-mimicking diet for critically ill patients: the pilot randomized crossover ICU-FM-1 study
Source: Crit Care. 2020 May 24;24:249. doi: 10.1186/s13054-020-02987-3 (PMC7245817; doi:10.1186/s13054-020-02987-3)
Supplement: Supplementary file 4 — Additional file 4. (VanDyck-ICU-FM-additional_table4). Cause of death for 90 day mortality. Description of data: Cause of death for patients who died within 90 days after randomization [file 13054_2020_2987_MOESM4_ESM.docx]

**Additional table 4: Cause of death for 90 day mortality**

| **Cause of death** | **Fasting – Feeding**  **N = 35** | **Feeding – Fasting**  **N = 35** |
| --- | --- | --- |
| **Overall 90 day mortality – n (%)** | **10 (28.6)** | **23 (65.7)** |
| Terminal malignancy – n (%) | 1 (6.7) | 3 (12.5) |
| Terminal interstitial lung disease – n (%) | 0 (0.0) | 2 (8.3) |
| Refractory cardiac failure – n (%) | 2 (13.3) | 2 (8.3) |
| Invasive aspergilosis – n (%) | 2 (13.3) | 0 (0.0) |
| Non resolving ARDS – n (%) | 1 (6.7) | 1 (4.2) |
| Non resolving ARDS and muscle weakness – n (%) | 0 (0.0) | 3 (12.5) |
| Weaning failure due to muscle weakness – n (%) | 1 (6.7) | 2 (8.3) |
| Generalized post-ICU muscle weakness – n (%) | 0 (0.0) | 1 (4.2) |
| Persistent GI fistula – n (%) | 0 (0.0) | 1 (4.2) |
| Ischemic/hemorrhagic stroke – n (%) | 0 (0.0) | 4 (16.7) |
| Post anoxemic irreversible brain damage – n (%) | 1 (6.7) | 2 (8.3) |
| TBI – n (%) | 1 (6.7) | 1 (4.2) |
| Meningitis/encephalitis – n (%) | 1 (6.7) | 1 (4.2) |

The cause of death for patients who died within 90 days after randomization was extracted from the patients’ hospital records.

Abbreviations: ARDS: acute respiratory distress syndrome, ICU: intensive care unit, GI: gastrointestinal, TBI: traumatic brain injury.
